# Pre-operative predicting factor in visual outcome after macular hole surgery

**DOI:** 10.12669/pjms.36.5.1995

**Published:** 2020

**Authors:** Lubna Feroz, Syed Fawad Rizvi, Saliha Naz, M. Tanweer Hassan Khan

**Affiliations:** 1 Dr. Lubna Feroz, MBBS, FCPS (Ophth). Ophthalmologist, LRBT Tertiary Teaching Eye Hospital, Korangi 21/2, Karachi, Pakistan; 2 Dr. Syed Fawad Rizvi, MCPS (Ophth), FCPS (Ophth). Chief Consultant Ophthalmologist, LRBT Tertiary Teaching Eye Hospital, Korangi 21/2, Karachi, Pakistan; 3 Dr. Saliha Naz, FCPS (Ophth), FCPS (VR). Consultant Ophthalmologist, LRBT Tertiary Teaching Eye Hospital, Korangi 21/2, Karachi, Pakistan; 4 Dr. M. Tanweer Hassan Khan, MBBS, FCPS (Ophth). Ophthalmologist, LRBT Tertiary Teaching Eye Hospital, Korangi 21/2, Karachi, Pakistan

**Keywords:** Best corrected visual acuity, Macular hole, Macular hole index, Visual outcome

## Abstract

**Objective:**

To determine the effectiveness of macular hole index (MHI) as a predicting factor of visual outcome after full thickness macular hole surgery.

**Methods:**

This quasi-experimental study was conducted at LRBT Free Base Eye Hospital, Karachi from January 2018 to March 2019. Total 45 eyes of 45 patients with full thickness macular hole (FTMH) underwent preoperative Best Corrected Visual Acuity (BCVA) assessment with logMar chart and Optical Coherence Tomography (OCT) scanning, with measurement of base diameter and macular hole height. Values were calculated for the macular hole index (MHI), which was taken as the predictive factor. All patients had undergone 25^+^G trans-conjunctival three ports pars plana vitrectomy, internal limiting membrane peeling, and endo-tamponade of C_3_F_8_(14%). The final visual outcome of all the patients was noted.

**Results:**

Forty-five patients were included for the study, out of which 10 (22.2%) were male and 35 (77.7%) were female. Age ranged from 45-70 years (mean age 57.20±6.47 years). The mean pre-operative visual acuity was 2.46±1.15 logMar and was 3.88±2.00 logMar, post-operatively. Moreover, 27(60.0%) out of 45 patients achieved BCVA (gain of 2 lines of the logMar chart). The average macular hole index was 1.55±0.50 and out of 45, 25 patients had MHI ≥0.5. It was found that patients with macular hole index ≥0.50 showed clinically significant improvement in BCVA in comparison to those who have macular hole index <0.50.

**Conclusion:**

Macular hole index can be used to predict functional success in macular hole surgery.

## INTRODUCTION

The prevalence of full thickness macular hole (FTMH) in the general population is estimated to be ≈3.3 per 1,000 people. It usually affects individuals in their sixth or seventh decade of life, and approximately two-thirds of them are females.^1^,^2^ Macular holes are classified into four stages, Stage-1 is known as the impending hole, while Stage-2 is a full thickness macular hole (FTMH) which measures <400µ, Stage-3 is FTMH of size ≥400µ and stage 4 is a ≥400µ hole with posterior vitreous detachment. Basic steps of macular hole surgery are vitrectomy, posterior vitreous cortex separation and intraocular gas tamponade, which was first described by Kelly and Wendel in 1991.^3^ Moreover, the addition of internal limiting membrane removal (ILM) was proposed by Eckardt et al. in order to improve the prognosis of macular hole surgery.^4^ This technique with internal limiting membrane peeling provides anatomical closure, even of large MH, in 90%-95%.^5^ Vital dyes are also used to facilitate visualization and safer removal of ILM during surgery, including the indocyanine green (ICG),^6^,^7^ brilliant blue G and the trypan blue.^8^

Recently, the use of optical coherence tomography (OCT) has proven to have an important role for diagnosing macular hole as well as for the pre-operative assessment of anatomical closure and visual success.^9^ There are two broad types of OCT, time domain and spectral domain, among which spectral domain has the better resolution and gives the 3D image of the retina. Spectralis OCT (Heidelberg Engineering) device works on the principle of spectral domain and measures the dimensions of macular hole such as minimum linear dimension, basal hole diameter and hole height. This machine has now become crucial in the preoperative diagnosis of macular hole,^10^ assessment of its dimensions, calculation of macular hole parameters such as macular hole index. The macular hole index represents the ratio between the height of macular hole and the diameter of its base, and it has been considered as indicative of better visual and anatomical prognosis, when the value equals to or greater than 0.5, and this is how it is useful in predicting the outcome of macular hole preoperatively.^11^ Apart from MHI there are other indices as well such as THI (tractional Hole Index) and HFF (hole form factor), which utilize different dimensions of macular hole for prediction of its surgical outcome. Nevertheless, in the present study, we are evaluating the relationship between the visual outcome of macular hole surgery with the value of macular hole index.

## METHODS

This was a quasi-experimental study which included 45 eyes of 45 patients, who presented with Stage-2, 3 and 4 macular holes. Patients were enrolled using non-probability consecutive sampling technique. Subject recruitment started from 1^st^ Jan, 2018 and was done till 30th Sep, 2018. Follow-up time was six months ending on 31^st^ Mar, 2019. The Hospital Ethics Committee approval was taken prior to the commencement of this study. Ref. No. LRBT/FBEH/ERC/019, dated: 28^th^ March, 2019.

Complete ocular history was taken to rule out any past ocular surgery, ocular trauma, past history of uveitis or any other ocular disease which can lead to a secondary macular hole. Patients with significant cataract (NS+3 and more, PSCC+2 or more), Stage-1 macular hole, macular cysts and secondary macular hole were excluded from the study. Only those patients with FTMH, Stage-2,3 and 4 and with no secondary causes, and those with NS+1 and pseudophakic were included in the study. Preoperative examination included best corrected visual acuity, complete slit-lamp examination, intraocular pressure assessment (applanation tonometer). Fundoscopy for posterior pole examination was done with 78D lens and peripheral retina was examine with Goldmann 3 mirror lens. Spectral Domain Optical Coherence Tomography (Spectralis, Heidelberg Engineering) was used to confirm the diagnosis and stage the macular hole, the measurements of macular hole parameters (hole height and basal hole diameter) were also performed to calculate the macular hole index. Written informed consent was taken from patients who fulfilled the criteria for the study, after the explanation of its benefits and possible worse consequences.

### Surgical Procedure

Surgery was performed under local peri-bulbar anesthesia, with a mixture of lidocaine (2%) and bupivacaine (0.7%),3-4ml and under all aseptic measures. Pars Plana Vitrectomy(25^+^gauge) was done using Constellation Vision System (Alcon surgicals®), by a single vitreoretinal surgeon. After completion of core vitrectomy, the posterior vitreous detachment was induced (if not already present). 0.5ml of ICG (indocyanine green) dye was used in the concentration of 0.125% to stain the ILM, and after staining, peeling of ILM around the macular hole was done in a circumferential manner by using end-grasping vitreoretinal forceps. An isovolumic concentration of C_3_F_8_ (14%) was injected at the end of the surgery after the air-fluid exchange. All patients were examined on the first postoperative day, first week, first month and then at sixth month. On every visit, complete ocular examination was performed, which included visual acuity using the logMar chart, anterior segment examination, IOP assessment, posterior pole evaluation, and assessment of macular hole closure using SD-OCT. Complications like overfilled gas tamponade and post-surgery cataract were recorded and managed accordingly. IBM (International Business Machines) SPSS (Statistical Package for Social Sciences) Statistics 21 was used to analyze the data. Frequencies with percentages were used to present qualitative variables and Mean±SD were calculated for the quantitative variables. Post-operative visual improvement was stratified with respect to macular hole index. Visual gain is considered when there is an increase in the visual acuity by two lines while there would consider to be no gain in vision if the visual acuity did not increase by two lines, using logMar chart. Paired t-test was applied to determine the significance of improvement in BCVA. P-value ≤ 0.005 was taken as significant.

## RESULTS

There were 45 patients with macular hole who underwent macular hole surgery with ILM peeling. The mean age was 57.20 ±6.47 years. Out of them, 10(22.2%) were males and 35(77.7%) females. The macular hole closure was achieved in 42(93.3%) eyes, whereas, 03(6.6%) of them remained unchanged. The patients presented with mean preoperative visual acuity of 2.46±1.15 logMar. The study showed that patients had a mean postoperative visual acuity of 3.88±2.00 logMar, among them 27(60.0%) eyes achieved BCVA (gain in two lines of the logMar chart). It also showed that pre-operatively most of the patient had poor visual acuity while postoperatively more patients attain better visual acuity, Table-I. Our study did not find any significant correlation between gender or age, and gain in visual acuity. The study supports that patients with better postoperative best corrected visual acuity, also had gain in visual acuity, Table-II. The average macular hole index was 1.55±0.50 and out of 45, 25(55.5%) patients had MHI≥0.5. Those patients who had poor visual acuity pre-operatively, among them, majority showed MHI of ≤0.5 and vice versa, Table-III. It was found that the patients with the macular hole index ≥0.5 showed clinically significant improvement in BCVA in comparison to those who had macular hole index <0.50 Table-IV, which was also statistically significant(p=0.004).

Correlation between pre-operative and post-operative best corrected visual acuity is shown in Table-I

**Table-I:** Correlation between pre-op and post-op best corrected visual acuity.

		POST-OP VA							Total

		CF	6/60	6/36	6/24	6/18	6/9	6/6	
PRE-OP VA	CF	8	1	2	0	1	0	0	12
	6/60	1	2	2	1	3	2	0	11
	6/36	0	1	0	1	7	3	0	12
	6/24	1	0	1	0	0	7	0	9
	6/18	0	0	0	0	0	0	1	1

Total	10	4	5	2	11	12	1	45	

Correlation between post-operative best corrected visual acuity and gain in visual acuity is shown in Table-II.

**Table-II:** Post-op BCVA and VA gain correlation.

		VA Gain		Total

		Gain	Not Gain	
POSTOPVA	CF	0	10	10
	6/60	0	4	4
	6/36	2	3	5
	6/24	1	1	2
	6/18	11	0	11
	6/9	12	0	12
	6/6	1	0	1

Total	27	18	45	

Correlation between pre and post-operative best corrected visual acuity with macular hole index is shown in Table-III.

**Table-III:** Correlation between pre-op and post-op BCVA with MHI.

POST-OP VA			PRE-OP VA					Total

			CF	6/60	6/36	6/24	6/18	
CF	MHI	<0.50	7	1		1		9
		>0.50	1	0		0		1
	Total		8	1		1		10
6/60	MHI	<0.50	1	2	1			4
	Total		1	2	1			4
6/36	MHI	<0.50	1	0		0		1
		>0.50	1	2		1		4
	Total		2	2		1		5
6/24	MHI	<0.50		0	1			1
		>0.50		1	0			1
	Total			1	1			2
6/18	MHI	<0.50	0	1	3			4
		>0.50	1	2	4			7
	Total		1	3	7			11
6/9	MHI	<0.50		1	0	3		4
		>0.50		1	3	4		8
	Total			2	3	7		12
6/6	MHI	>0.50					1	1
	Total						1	1
Total	MHI	<0.50	9	5	5	4	0	23
		>0.50	3	6	7	5	1	22
	Total		12	11	12	9	1	45

Table-IV Shows macular hole index with respect to the best corrected visual acuity where it can be seen that majority of the patients who gain the best corrected visual acuity had macular hole index of ≥0.5. However, among those who did not gain best corrected visual acuity, many of them had macular hole index <0.5.

**Table-IV:** MHI regarding POST-OP BCVA.

POST-OP BCVA	MHI		Total

	<0.50	≥0.50	
Gain	09	18	27
No Gain	14	04	18

Total	23	22	45

Coefficient of correlation= 0.003.

## DISCUSSION

Macular hole size and its dimensions hold significant importance in predicting the results of macular hole surgery.^12^ Therefore, their measurements are crucial to determine surgical success.^13^ The introduction of optical coherence tomography (OCT) has allowed a more accurate visualization of its anatomy, measurement and better knowledge of the pathophysiology of the process.^14^ It is widely used in the measurement and assessment of MH characteristics like MHI, facilitating treatment, decision-making and the expected surgical outcome.^15^ There are numerous studies that have utilized the OCT parameters obtained from MH eyes to predict the visual outcomes such as macular hole size, hole height, basal hole diameter. Among them, it is proposed that the macular hole index (MHI) is an important predictor for visual outcome following MH surgery.^16^-^18^ The MHI is defined as the ratio of the hole height to the basal hole diameter and is reported to be positively correlated to the postoperative visual acuity in several studies.^19^ It is also found that eyes with an MHI value ≥0.5 had better visual acuity than those with an MHI value <0.5.^20^,^21^

The macular hole index that can reflect the prognosis of the surgery and its visual outcome has been gaining popularity among the publications on the macular hole and it’s now been used as a useful predictor for the physiological outcome of MH.^22^,^23^ The determination of the macular hole index as was obtained in this study are of easy application, therefore, the better outcome of the surgical procedure can be pre-operatively determined. The functional results found in this study are comparable to those of literature,^24^,^25^ showing that MHI is a good predictive factor for FTMH surgery outcome. We did not find any correlation between gender and age with the outcome of macular hole surgery. However, the small sample is a limitation of this study as there is a lower prevalence of the disease.

## CONCLUSION

An MHI ≥ 0.50 is an effective predictive factor for a good visual prognosis after Idiopathic Macular Hole surgery.

### Authors’ Contribution

**LF:** Patient Clinical Assessment and OCT conductor. She is also responsible and accountable for the accuracy or integrity of the study.

**SFR:** Senior Vitreoretinal Surgeon who performed the surgery.

**SN:** Data Collection and Recording.

**THK:** Data Collection and Recording.
